# Opioids and Viral Infections: A Double-Edged Sword

**DOI:** 10.3389/fmicb.2016.00970

**Published:** 2016-06-22

**Authors:** Alireza Tahamtan, Masoumeh Tavakoli-Yaraki, Talat Mokhtari-Azad, Majid Teymoori-Rad, Louis Bont, Fazel Shokri, Vahid Salimi

**Affiliations:** ^1^Department of Virology, School of Public Health, Tehran University of Medical SciencesTehran, Iran; ^2^Department of Biochemistry, School of Medicine, Iran University of Medical SciencesTehran, Iran; ^3^Department of Pediatrics, Wilhelmina Children's Hospital, University Medical Centre UtrechtUtrecht, Netherlands; ^4^Department of Immunology, School of Public Health, Tehran University of Medical SciencesTehran, Iran

**Keywords:** opioids, opioid receptors, immunomodulation, viral infections, therapies, immune responses, opioid signaling, anti-inflammation

## Abstract

Opioids and their receptors have received remarkable attention because they have the ability to alter immune function, which affects disease progression. *In vitro* and *in vivo* findings as well as observations in humans indicate that opioids and their receptors positively or negatively affect viral replication and virus-mediated pathology. The present study reviews recent insights in the role of opioids and their receptors in viral infections and discusses possible therapeutic opportunities. This review supports the emerging concept that opioids and their receptors have both favorable and unfavorable effects on viral disease, depending on the type of virus. Targeting of the opioid system is a potential option for developing effective therapies; however caution is required in relation to the beneficial functions of opioid systems.

## Opioids and their receptors

Opioids are a group of endogenous and exogenous/synthetic compounds that function through activation of opioid receptors (McNally and Akil, 2002). Opioid receptors have been isolated, cloned, and classified as μ-opioid receptors (MORs), δ-opioid receptors (DORs), κ-opioid receptors (KORs), and opioid-like receptors (ORL), and are encoded by OPRM1, OPRD1, OPRK1, and OLR genes, respectively (Stevens, 2009). The MOR (μ1, μ2, μ3), DOR (δ1, δ2), and KOR (κ1, κ2, κ3) opioid receptor genes have been cloned (Herz, 1998). Opioid receptors are mainly distributed in the central and peripheral nervous system, as well as in non-neuronal cells in the lung, spleen, liver, intestine, adenoid, and kidney (Wittert et al., 1996).

The receptors are transmembrane G protein-coupled receptors (GPCRs) with an affinity for endogenous opioid peptides and include β-endorphin, encephalin, met-enkephalin, nociceptin, and exogenous natural and synthetic opioid agents (e.g., morphine, heroin; Stein, 2016). A high degree of structural similarity (50–70%) has been observed in the transmembrane domains 2, 3, and 7 and the first and second intracellular loops. The extracellular domain diverges, which explains differences in ligand selectivity between opioid receptors (McNally and Akil, 2002). Following activation of the opioid receptors, adenylyl cyclase is inhibited, which activates K^+^ channels and diminishes the conductance of voltage-sensitive Ca^2+^ channels (Stein, 2016). Opioid receptors also activate mitogen-activated protein kinases (MAPK) and phospholipase C-mediated signaling, leading to the formation of IP3 and diacyl glycerol (Childers, 1991). All these effects lead to activation or inhibition of several downstream signals that contribute to the intrinsic effects of opioids (Stein, 2016).

## Opioids and immune function

**Figure 1:**
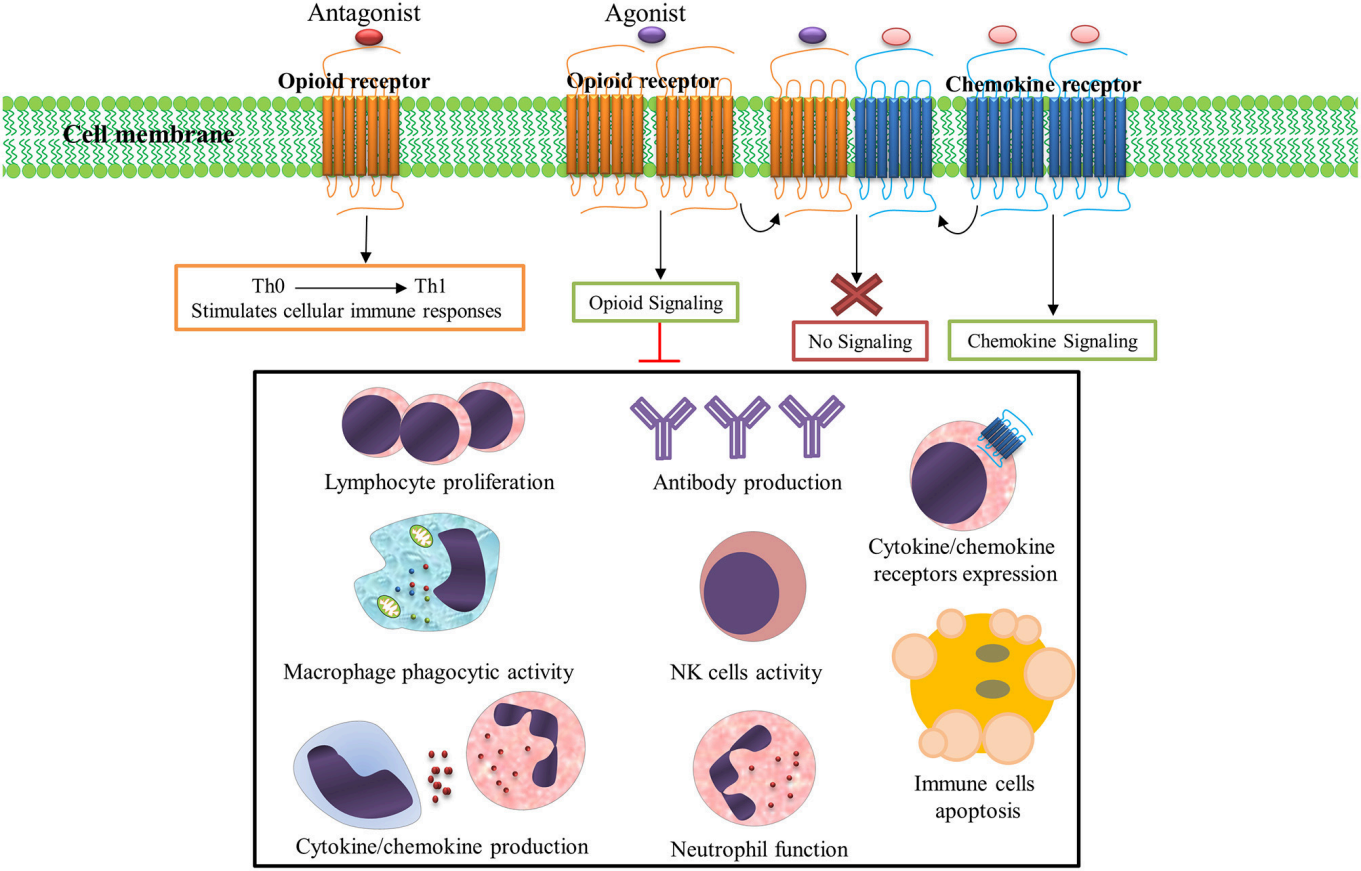
**Opioid systems effects on immune responses**. Opioid receptors are abundantly expressed on various immune cells and immune function is influenced by these receptors activation/inhibition. Numerous immune factors and immune responses are significantly dampened following opioid receptors activation. Opioid and chemokine receptors interaction on the immune cells surface regulates immune responses; Activation of opioid receptors by their ligands desensitizes chemokine receptors and also activation of chemokine receptor by its ligand desensitizes opioid receptor. Importantly, opioid receptors antagonists stimulates cellular immune responses and shifts immune responses to a Th1 pattern.

Opioids and their derivatives are the oldest and most effective drugs for treatment of pain (Lewis et al., 1987). They are also one of the most popular recreational drugs and account for 27 million users worldwide (UNODC, 2015). Opioid-mediated analgesia occurs through modulation of ascending and descending pain pathways (Kapitzke et al., 2005). The immunomodulatory and anti-inflammatory properties of opioids have also been well documented (Bidlack, 2000). Opioid receptors are abundantly expressed on various immune cells (e.g., lymphocytes, macrophages, neutrophils, and monocytes) and it has been shown that immune function is influenced by these receptors (Figure 1; McCarthy et al., 2001; Odunayo et al., 2010). It has been shown that immune cells contain opioid peptides such as β-endorphin, metenkefalin, and dynorphin A, which have been proposed to alleviate inflammatory hyperalgesia (Kapitzke et al., 2005).

Numerous immune factors and immune responses are significantly dampened following opioid exposure in drug users, healthy human subjects, and animal models (Rogers and Peterson, 2003; Sacerdote, 2006). These dampened immune functions include suppressed cytokine/chemokine production (Finley M. et al., 2008), cytokine/chemokine receptors expression (Finley M. J. et al., 2008), antibody production (Taub et al., 1991), lymphocyte proliferation (McCarthy and Rogers, 2001), NK cell activity (Shavit et al., 1984), neutrophils function (Simpkins et al., 1986), and macrophage phagocytic activity (Rojavin et al., 1993). The activation of opioid receptors also induces apoptosis in immune cells (Singhal et al., 1998); thus, endogenous/exogenous opioids, opioid receptors, and opioid signaling pathways together form a system (opioid system) which modulates immune function (Salimi et al., 2013).

Opioid receptors and chemokine receptors and their ligands are widely expressed in brain tissue and immune cells (McCarthy et al., 2001). Multiple lines of evidence have shown that interaction between opioids and chemokine receptors regulate immune responses (Steele et al., 2002). Potential mechanisms for this interaction include heterologous desensitization (also known as cross-desensitization), changes in receptor expression, opioid and chemokine receptor dimerization or heteromerization, and regulation by the ferritin heavy chain (Rogers et al., 2000; Nash and Meucci, 2014). Activation of GPCRs such as opioids on the surface of immune cells often inhibits chemokine receptors by heterologous desensitization. The inhibitory crosstalk between opioids and chemokine receptors is believed to be a major element in regulation of the function of these receptors (Ali et al., 1999; Rogers et al., 2000). Several forms of desensitization occur between opioid and chemokine receptors. MOR and DOR are able to induce heterologous desensitization of CCR1, CCR2, CCR5, CXCR1, and CXCR2 (Grimm et al., 1998). The CCR5 chemokine receptor can form heterodimers with the opioid receptors MOR, DOR, and KOR (Steele et al., 2002; Szabo et al., 2003; Chen et al., 2004). KOR is able to cross-desensitize CXCR4 (Finley M. J. et al., 2008) and ORL1 induces desensitization of CXCR4 (Kaminsky and Rogers, 2011). Simultaneous stimulation of CXCR4 and DOR-expressing cells by their respective ligands, CXCL12 and DPDPE, does not trigger receptor function (Pello et al., 2008). Opioid and CCR5/CXCR4 interaction potentially contribute to HIV-associated neurocognitive disorders (Steele et al., 2003).

Importantly, besides immunomodulatory and anti-inflammatory activity, the opioid system could modulate T-helper (Th) cytokine patterns (Sacerdote et al., 1998). Opioid receptor antagonists stimulate cellular immune responses and shifts immune responses to a Th1 pattern (Sacerdote et al., 2000; Jazani et al., 2011; Khorshidvand et al., 2016). This ability to induce and shift immune responses toward a Th1 pattern makes opioid receptors antagonists as a new adjuvant candidate in the induction of cellular immunity which could increase protection against intracellular parasites (Khorshidvand et al., 2016). These studies support the administration of opioid receptor blockers such as naloxone and/or naltrexone as a vaccine adjuvant to increase vaccine efficacy by enhancing cellular immunity (Jamali et al., 2009; Jazani et al., 2010, 2011; Hajipirloo et al., 2014; Shahabi et al., 2014; Khorshidvand et al., 2016). Taken together, the inhibition of anti-inflammatory and immunomodulatory activity of opioid signaling by opioid receptors antagonists explains the mechanisms by which opioid antagonists trigger vaccine-induced immunity (Khorshidvand et al., 2016). Although many advances have been made in understanding the effects of opioids system on immune regulation, it is necessary to understand the real clinical relevance of this system (Ninkovic and Roy, 2013).

## Opioids and viral infection

Although intensive research has rapidly advanced knowledge about the effect of the opioid system on immune function, little is known about the role of this system in the initiation and development of infection (Risdahl et al., 1998). Through activation of opioid receptors, opioid systems effect diverse biological functions, including both innate and adoptive immune responses which can affect susceptibility to pathogens (Wang et al., 2008). It is well documented that opioid abusers have a higher prevalence of opportunistic infections, which may be directly associated with impaired immune function (Roy et al., 2011). Studies in healthy non-addicted individuals has revealed that opioid treatment in obstetrics results in reactivation of latent viruses, which can be directly explained by a dampened antiviral immune function (Crone et al., 1988). A previous study by the authors indicates that endogenous opioids can control viral infection and could be useful for therapeutic applications (Salimi et al., 2013).

**Figure 2:**
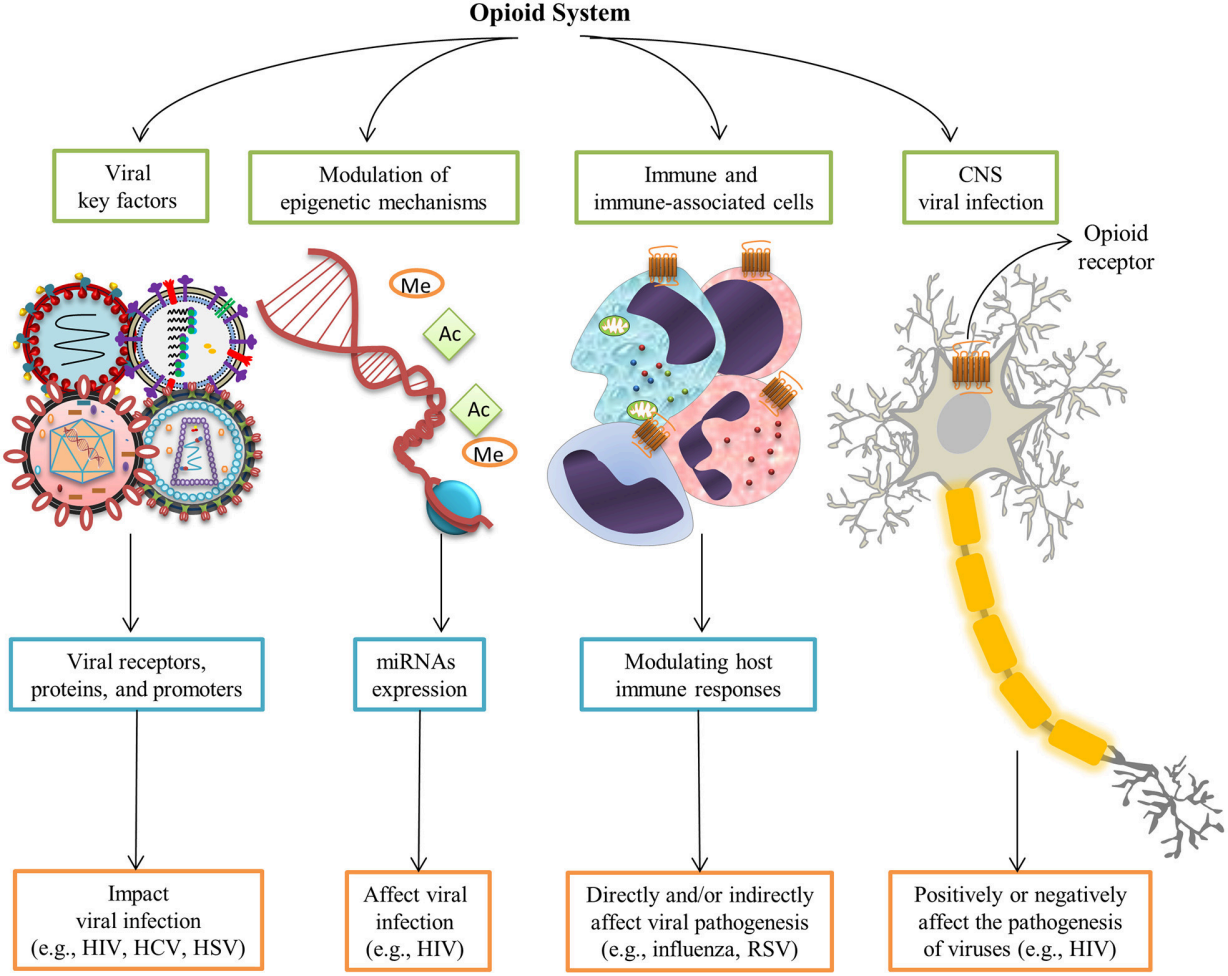
**Opioid systems effects on viral replication and pathogenesis**. Opioids may positively or negatively affect the pathogenesis of viruses directly through the central nervous system (Hu et al., 2011). Opioid receptors activation in immune and immune-associated cells directly or indirectly affect viral pathogenesis via modulating host immune responses (Salimi et al., 2013). Modulation of epigenetic mechanisms such as miRNAs expression affect viral infection (Purohit et al., 2012). Furthermore, interaction with several viral key factors such as viral receptors, viral proteins, and viral promoters may influence viral replication and virus-medicated pathology (Sundar et al., 1996; Steele et al., 2003; Chang and Connaghan, 2012).

Given the breadth of opioid-mediated signaling and immune regulation in mammals, the role of the opioid system has recently been highlighted in viral infections. The effects of opioids and their receptors have been studied for several viral infections (Table 1). Opioid systems affect viral replication and pathogenesis in several ways (Figure 2). Interestingly, opioids have either a beneficial or deleterious effect on the pathogenesis of viruses directly through central nervous system (CNS) by their intrinsic immunomodulatory actions (Hu et al., 2011). Depending on the virus type, activation of opioid receptors in immune and immune-associated cells directly or indirectly enhances (Wang C.-Q. et al., 2005) or suppresses viral pathogenesis by modulating host immune responses (Coussons-Read et al., 1999; Salimi et al., 2013). Findings on the ability of opioids to modulate epigenetic mechanisms, in particular microRNAs (miRNAs) expression, shed new light on possible mechanisms underlying the effect of opioids on viral infection (Purohit et al., 2012; Pilakka-Kanthikeel and Nair, 2015). Furthermore, opioid interaction with viral receptors, viral proteins, and viral promoters effect viral replication during the course of an infection (Sundar et al., 1996; Steele et al., 2003; Chang and Connaghan, 2012).

**Table 1:** **Opioid system effects on viral infections**.

**Viral infection**	**Effects**	**References**
HIV 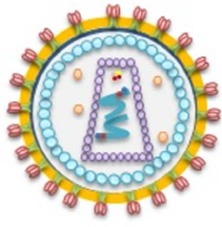
	Opioids impair the anti-HIV activity of immune system	Wang X. et al., 2005
	MORs activation up-regulates the CCR5 and CXCR4 expression	Steele et al., 2003
	Opioids induce the pro-inflammatory mediators expression	El-Hage et al., 2006
	Opioid system disrupts the gut homeostasis	Meng et al., 2015
	Opioids inhibit the anti-HIV miRNAs expression	Wang et al., 2011
	Opioid system reactivate the HIV latently infection	Prottengeier et al., 2014
	Opioids induce the cytokine production and HIV promoter activation	Sundar et al., 1996
	Morphine alters the β-chemokines and CCR5 receptor expression	Li et al., 2003a
	Morphine limits the ability of neurons to recover from injury	Masvekar et al., 2014
	Morphine promotes the growth of HIV in human PBMC	Peterson et al., 1990
	Morphine exacerbates the neuropathogenesis process of HIV-1 gp 120	Mahajan et al., 2005
	Morphine increases production of reactive free radical & oxidative stress	Turchan-Cholewo et al., 2009
	KORs activation suppresses HIV-1 expression in infected microglial	Chao et al., 1996
	KORs activation suppresses HIV-1 expression in human brain cells	Chao et al., 1998
	KORs activation inhibits HIV-1 expression in infected CD4 cells	Peterson et al., 2001
	KORs activation reduces HIV-1 entry	Lokensgard et al., 2002
	DORs activation in T cells reduces the expression of HIV-1	Sharp et al., 1998
	DORs activation attenuates Tat-induced intracellular oxidative stress	Wallace et al., 2006
	MORs activation reduces HIV-1 replication in TF-1 cells	Strazza et al., 2014
	Opioids suppress IL-16-induced signaling pathways	Chao et al., 1995
HCV 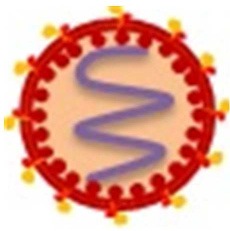
	Opioid dependence therapy associates with lower incidence of HCV	Tsui et al., 2014
	Morphine compromises anti-HCV effect of recombinant IFN-α	Li et al., 2003b
	Morphine inhibits intracellular IFN-α expression	Li et al., 2007
	Morphine withdrawal inhibits expression of endogenous IFN-α	Wang C.-Q. et al., 2005
	Opioids impair CD56+ T cell-mediated innate immune function	Ye et al., 2010
	Morphine induces hepatic pro-inflammatory cytokine and free radicals	El-Hage et al., 2011
	Opioid system synergize the alcohol acceleration of HCV expression	Zhang et al., 2003
	β-endorphins down-modulates T cell anti-bacterial response	Amati et al., 2001
	Met-enkephalin enhances replication of HCV and interferes with interferon	Bergasa and Boyella, 2008
	Met-enkephalin as a marker in hepatocellular damage in chronic HCV	Ciesla et al., 2006
Influenza 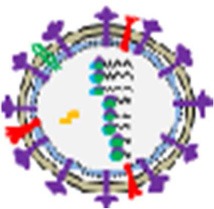
	Morphine impairs the inflammatory response to influenza in the lungs	Hu et al., 2011
	Morphine treated rates slowly clear virus from their lungs	Coussons-Read et al., 1998
	Opioid system modulate NK cell cytotoxicity during influenza infection	Tseng et al., 2005
	Opioids could increase risk of pneumonia after influenza as a consequent of immune suppression	Dublin et al., 2011
	Exposure to methadone significantly increased H1N1 viral replication	Chen et al., 2015
RSV 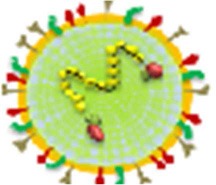
	The A118G single nucleotide polymorphism rs1799971 associated with RSV disease severity	Salimi et al., 2013
	Opioid system control RSV replication in the lung and consequently control virus immunopathogenesis	Salimi et al., 2013
HSV 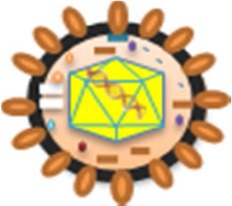
	Morphine alters innate immune responses against HSV-1	Sheridan and Moynihan, 2005
	Morphine diminishes protective innate immune defense against HSV-1	Jamali et al., 2007a
	Morphine reduced CTL responses, lymphocyte proliferation, and IFN-γ	Mojadadi et al., 2009
	Withdrawal from morphine reduces protective immunity against HSV-1	Jamali et al., 2012
	Endogenous opioids could suppress protective immunity against HSV-1	Jamali et al., 2007b
	Morphine treatment reduces HSV-1 mortality in infected mice	Alonzo and Carr, 1999
	Morphine treatment reduces HSV-1 pathogenesis in infected mice	Weeks et al., 2001
	High incidence of HSV in patients given epidural morphine	Gieraerts et al., 1987
	Epidural morphine reactivates oral herpes in the obstetric population	Crone et al., 1990
	Morphine potentiates development of encephalitis in HSV-1 infected mice	Lioy et al., 2006
	Attenuated hippocampal dynorphin causes seizures in HSV-1 infected rats	Solbrig et al., 2006
	MORs activation by loperamide suppress mechanical allodynia in mice with herpetic pain	Sasaki et al., 2007
	MORs activation by morphine suppress mechanical allodynia in mice with herpetic pain	Sasaki et al., 2008

The present study summarizes and discusses the effect of the opioid system on human viral infections, which are amongst the most important human pathogens. It is proposed that opioid system activity increases during viral infection and is involved in viral pathogenesis. Understanding the role of opioid system in viral infection is of importance for understanding disease pathogenesis and could lead to novel therapeutic approaches for viral infections.

### Human immunodeficiency virus

**Figure 3:**
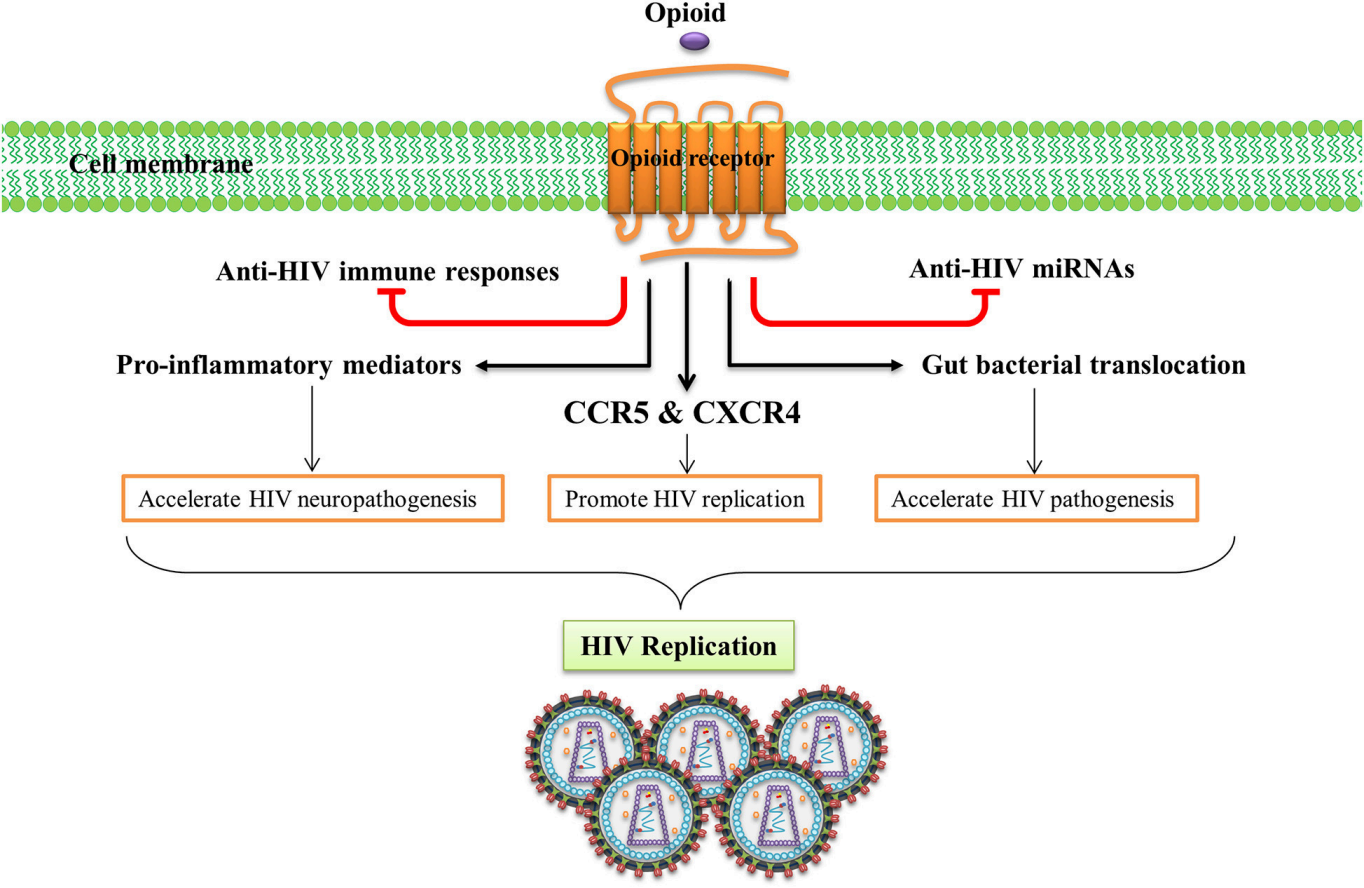
**Opioid systems effects on HIV replication and pathogenesis**. Opioid system accelerate HIV pathogenesis and enhance susceptibility to HIV via several mechanisms. Opioid system impair the anti-HIV activity of immune system (Wang X. et al., 2005), inhibit the expression of anti-HIV miRNAs (Wang et al., 2011), induce the expression of several pro-inflammatory mediators (El-Hage et al., 2006), up-regulate the expression of HIV-1 co-receptors (Steele et al., 2003), disrupt gut homeostasis by modulating gastrointestinal motility and suppressing intestinal immune functions leading to enhanced gut bacterial translocation and disease progression during HIV infection (Meng et al., 2015).

Human immunodeficiency virus (HIV) causes acquired immunodeficiency syndrome (AIDS), a serious cause of death in humans of all ages (Maartens et al., 2014). Many HIV-infected subjects abuse morphine, heroin, or other opioid-related substances (Wang and Ho, 2011). Nearly half of those problematic drug users inject drugs and an estimated 1.65 million of IV drug users were living with HIV in 2013 (UNODC, 2015). Opioids have been suggested as promoting the pathogenesis of HIV in the CNS and exacerbating neurodegenerative diseases caused by chronic HIV (Hauser et al., 2005). In addition, HIV-infected opioid abusers are at higher risk of developing HIV dementia (Turchan-Cholewo et al., 2008). Importantly, accumulating evidence from *in vitro* and *in vivo* studies indicates that the opioid system can act as a cofactor in the immunopathogenesis of HIV, as they have the potential to impair host immune response and enhance microbial infections (Rouveix, 1992). Several mechanisms have been proposed for the peripheral and neurotoxic effects of opioids in HIV infection, viral replication, and disease progression (Figure 3).

The effect of opioids on the immune system plays an essential role in HIV peripheral and CNS pathogenesis. Opioid administration impairs the anti-HIV activity of the immune system which enhances HIV pathogenesis (Wang X. et al., 2005). Activation of opioid receptors, especially MOR, up-regulates expression of the chemokine receptors CCR5 and CXCR4, two major HIV-1 entry co-receptors, which promotes HIV-1 replication (Steele et al., 2003). Opioids induce expression of several pro-inflammatory mediators such as tumor necrosis factor-α (TNF-α), interleukin (IL)-6, and CCL5 and accelerates HIV neuropathogenesis (El-Hage et al., 2006). Opioids have been shown to disrupt gut homeostasis by modulating gastrointestinal motility and suppressing intestinal immune functions elevating gut bacterial translocation, which activates the immune system and accelerates disease progression during HIV infection (Meng et al., 2015). Endogenous opioids, as do exogenous opioids, can potentiate HIV-1 replication by inducing cytokine production and stimulation of HIV promoter (Sundar et al., 1996).

Morphine is a widely-used opioid that accelerates HIV peripheral pathogenesis in several ways. Morphine treatment enhances susceptibility of neonatal monocyte-derived macrophages to HIV infection most likely through alteration of β-chemokines and CCR5 receptor expression (Li et al., 2003a). Morphine promotes growth of HIV-1 in human peripheral blood mononuclear cells by altering the function of human T lymphocytes and monocytes (Peterson et al., 1990). Furthermore, HIV-1 gp120 primes lymphocytes for morphine-mediated apoptosis (Moorman et al., 2009).

While the opioid system can act as a cofactor in the pathogenesis of HIV, in some instances it can also inhibit HIV expression. Chao et al. reported that activation of KORs suppresses HIV-1 expression in infected microglial (Chao et al., 1996) and in human brain cells by potentiation of TNF-α (Chao et al., 1998). Activation of KORs inhibits expression of HIV-1 in infected CD4 lymphocyte cultures (Peterson et al., 2001) and reduces HIV-1 entry through inhibition of envelope gp-mediated membrane fusion and CXCR4 expression (Lokensgard et al., 2002). Sharp et al. have shown that activation of DORs in T cells reduces the expression of HIV-1. Activation of DORs affects intracellular signaling that is known to mediate CD4 T cell activation and expression of HIV-1 (Sharp et al., 1998). DOR activation also attenuates Tat-induced intracellular oxidative stress in SK-N-SH cells (Wallace et al., 2006). Strazza et al. stated that MOR activation by DAMGO alters expression of CXCR4 and reduces HIV-1 replication in TF-1 human bone marrow progenitor cells (Strazza et al., 2014). Furthermore, opioids suppress IL-16-induced signaling pathways, which in turn inhibit HIV-1 expression in chronically infected promonocyte clone 1 (Chao et al., 1995).

The effect of opioids on epigenetics, especially miRNA expression, could affect HIV replication and pathogenesis. For example, the opioid system inhibits expression of anti-HIV miRNAs (e.g., miRNA-28, 125b, 150, and 382) that target 3′-UTR of HIV transcripts (Wang et al., 2011). Interestingly, Purohit et al. found that down-regulation or inhibition of the activity of this miRNA can stimulate replication of latent HIV-1 in resting CD4^+^ T cells and monocytes (Purohit et al., 2012). Opioids also reactivate latent HIV infections *in vitro* which is associated with activation of reactive oxygen species (ROS) and nuclear factor-kappa B (NF-κB) (Prottengeier et al., 2014).

Differential expression of alternatively-spliced opioid receptor isoforms and polymorphisms in HIV-infected subjects could affect the pathophysiology of HIV infection (Dever et al., 2014). Proudnikov et al. showed that OPRM1 polymorphism alters the severity of HIV infection before and after HAART (Proudnikov et al., 2012). They also revealed that OPRK1 and PDYN polymorphism alters the severity of HIV infection and response to treatment (Proudnikov et al., 2013).

Morphine increases neurotoxic effects of HIV, and also limits the ability of neurons to recover from sublethal damage (Masvekar et al., 2014). Also, morphine synergizes the toxic effect of HIV proteins. For example morphine exacerbates neuropathogenesis of HIV-1 glycoprotein (gp) 120 by altering the chemokine balance in the brain (Mahajan et al., 2005). Importantly, HIV-1 gp120 and morphine cooperatively induce oxidative stress and affect cell cycle machinery, which may cause disease progression (Samikkannu et al., 2015). The interaction of the HIV-1 transactivator of transcription (Tat) and morphine increases the neurodegenerative effects through MORs signaling (Zou et al., 2011).

Turchan-Cholewo et al. showed that Tat and morphine synergistically increase the production of ROS (Turchan-Cholewo et al., 2009). HIV-Tat triggers opioid signaling in the microglia through induction of receptor expression or alters ligand-induced trafficking of opioid receptors (Turchan-Cholewo et al., 2008). Therapeutic interventions that target the opioid system could be targeted to mitigate neuropathogenesis and neuroAIDS. For example, Sagar et al. demonstrated that using a novel therapeutic approach (neurotargeting via magnetic nanocarrier) could be effective in targeting opiate-induced neuropathogenesis and neuroAIDS (Sagar et al., 2015). Yuan et al. suggested that a properly designed bivalent ligand targeting the putative MOR and CCR5 heterodimers could block HIV invasion into host cells by specifically targeting the putative MOR-CCR5 heterodimer (Yuan et al., 2013).

The data indicates that opioid systems have a significant effect on HIV infection, pathogenesis, and immune regulation. Mechanisms associated with enhancement of HIV pathogenesis through the opioid system appear to work through activation of opioid receptors and their downstream signaling pathways to make them possible targets for therapeutic intervention in HIV infection.

### Hepatitis C virus

The hepatitis C virus (HCV) causes severe liver disorders such as fibrosis, cirrhosis, and cancer (Webster et al., 2015). HCV prevalence is high among opioid-dependent individuals (range 10–90% in different region; UNODC, 2015), and HCV-related disorders are common causes of morbidity and mortality in opioid users (Gibson et al., 2011; Nelson et al., 2011). Opioid users are exposed to needle sharing, unsafe disposal and unsuitable cleaning of needles, as well as unsafe sex, which make them prone to HCV infection (Hagan et al., 2001). Maintenance of opioid agonist therapy (methadone or buprenorphine) in opioid users is associated with lower incidence of HCV, which may be an important strategy for prevention of the spread of HCV among drug users (Tsui et al., 2014). Furthermore, simultaneous targeting of HCV infection and opioid dependence has been shown to be effective for drug users infected with HCV (Murphy et al., 2014). Despite the high prevalence of HCV in opioid users, little is known about molecular mechanisms contributing to HCV infection among opioid-dependent individuals.

**Figure 4:**
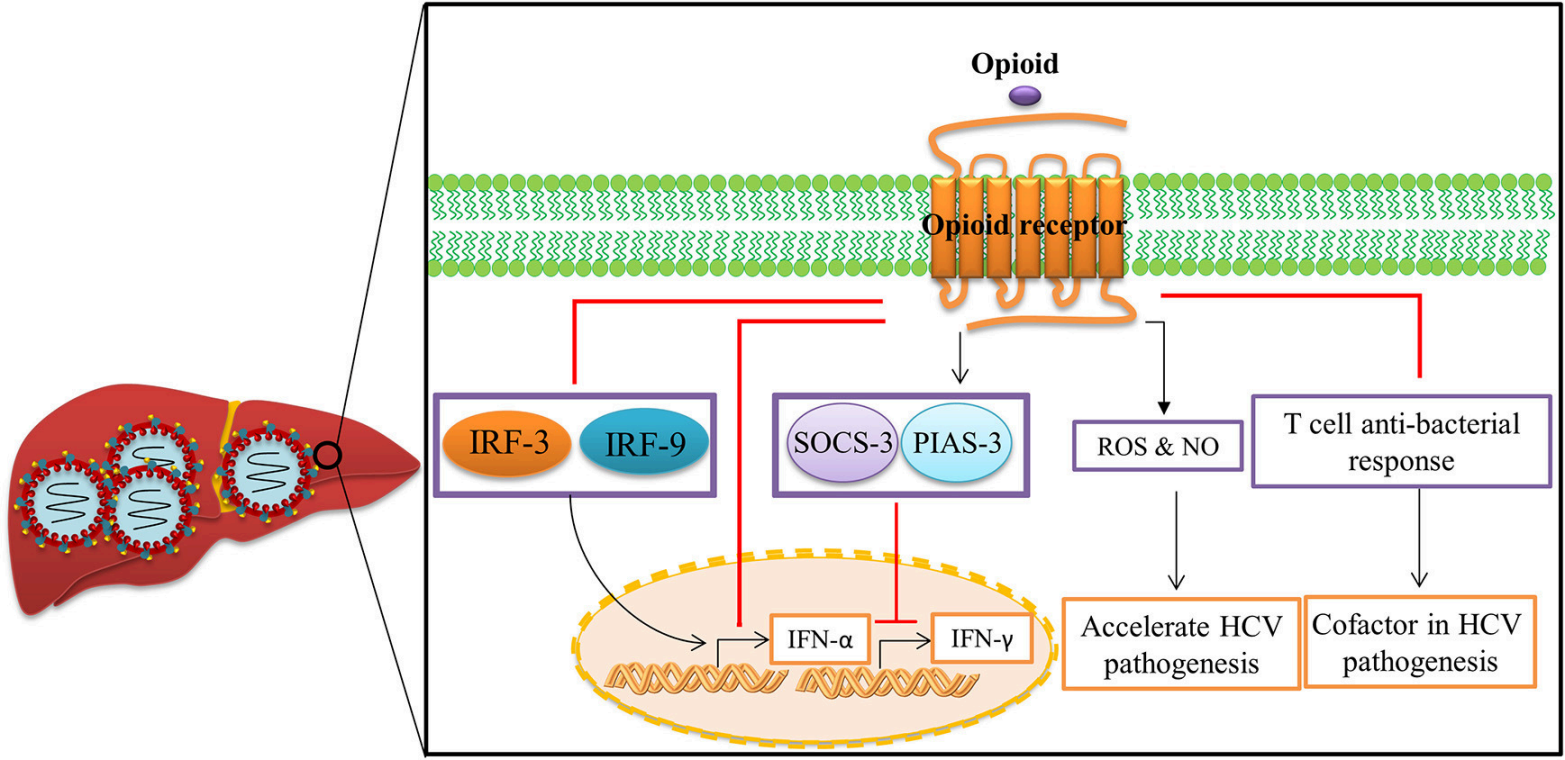
**Opioid systems effects on HCV replication and pathogenesis**. Opioid systems accelerate HCV pathogenesis and enhance susceptibility to HCV infection via several mechanisms. Opioid system impair the expression of IFN-α (Li et al., 2007), and IFN-γ (Ye et al., 2010), and also down-regulate the T cell anti-bacterial response in HCV patients by activation of opioid receptors in immune cells, which accelerates disease progression (Amati et al., 2001). Opioids also induce the expression of ROS and NO leading to disease progression (El-Hage et al., 2011).

Chronic opioid abuse has a severe negative impact on the immune system (Figure 4). The negative impact of opioids on host immunity in an important factor in the development of acute and chronic HCV infections (Zhang et al., 2006). *In vitro* investigations indicate that opioids facilitate HCV infection in hepatocytes by suppressing interferon (IFN)-α-mediated intracellular innate immunity (Li et al., 2003b, 2007; Wang C.-Q. et al., 2005). Li et al. demonstrated that morphine compromised the anti-HCV effect of recombinant IFN-α augmenting HCV replicon expression in hepatic cells (Li et al., 2003b). They revealed that morphine inhibited intracellular IFN-α expression in newly-infectious infected cells, which further facilitates HCV persistence in human hepatocytes (Li et al., 2007). Wang et al. reported that morphine withdrawal, a recurrent event during the course of opioid abuse, inhibits expression of endogenous IFN-α and enhances HCV replicon expression in hepatic cells (Wang C.-Q. et al., 2005). Morphine inhibits IFN-α by direct interaction with IFN-α promoter and interferon regulatory factors (IRF)-5, IRF-7, and p38 (Li et al., 2003b, 2007; Wang C.-Q. et al., 2005). These important *in vitro* findings were confirmed by Ye et al., who demonstrated that administration of opioids results in impairment of CD56^+^ T cell-mediated innate immune function (secretion of IFN-γ) through up-regulation of cytokine signaling 3 and protein inhibition of activated STAT 3 (SOCS-3), which may contribute to HCV infection and persistence in the liver (Ye et al., 2010).

Opioid-induced oxidative damage has been assumed to contribute to many of the systemic signs of liver disorder and toxicity as illustrated in mice as well as opioid abusers (Tennant and Moll, 1995; Payabvash et al., 2006). El-Hage et al. have shown that morphine accelerates HCV-mediated pathology by dysregulation of HCV-induced hepatic pro-inflammatory cytokines expression such as TNF-α and CCL5 and free radical production such as ROS and nitric oxide (NO) in HCV JFH1-infected hepatocytes (El-Hage et al., 2011). Furthermore, opioid system synergizes with the effect of alcohol to accelerate HCV replication, because enhancement of the effect of alcohol on HCV replicon expression was abrogated following administration of naltrexone as an opioid receptor antagonist (Zhang et al., 2003).

Endogenous opioids, like exogenous opioids, act as cofactors in HCV replication and pathogenesis. For example, β-endorphins down-modulate T cell anti-bacterial response in HCV patients by activation of opioid receptors in immune cells (Amati et al., 2001). The met-enkephalin concentration is high in the liver of HCV patients and is a useful marker of hepatocellular damage in chronic HCV (Ciesla et al., 2006). Met-enkephalin activates MORs and DORs which enhance replication of HCV in hepatic cells (Bergasa and Boyella, 2008), particularly in the liver of HCV patients (Boyella et al., 2008), and also interferes with the anti-viral effect of type I interferon (Floreani et al., 2012). The low efficiency of interferon therapy for HCV treatment in some patients could be explained by the effects of hepatic met-enkephalins (Bergasa and Boyella, 2008; Boyella et al., 2008).

Taken together, these results suggest that opioids accelerate HCV pathogenesis and establish chronic HCV infection, especially in drug abusers. Further consideration should be given to opioid abuse as a contributing factor for the increase in HCV infection among opioid-dependent individuals. Better understanding of the relationship between HCV and the opioid system may yield new therapeutic intervention in HCV carriers.

### Influenza virus

Opioid systems effect infections caused by HIV and HCV, but little is known about the role of opioid systems in the pathogenesis of other clinically important viral pathogens. Evaluating the effects of the opioid system on influenza infection could provide valuable information as it is a serious public health. Influenza causes viral respiratory tract diseases ranging from mild upper respiratory infection to severe pneumonia (Short et al., 2014). When airway epithelial cells are infected by the influenza virus, infiltration of various immune cells occurs. It is known that infiltrating immune cells are directed against infected epithelial cells, causing immunopathogenesis (Tahamtan et al., 2016).

**Figure 5:**
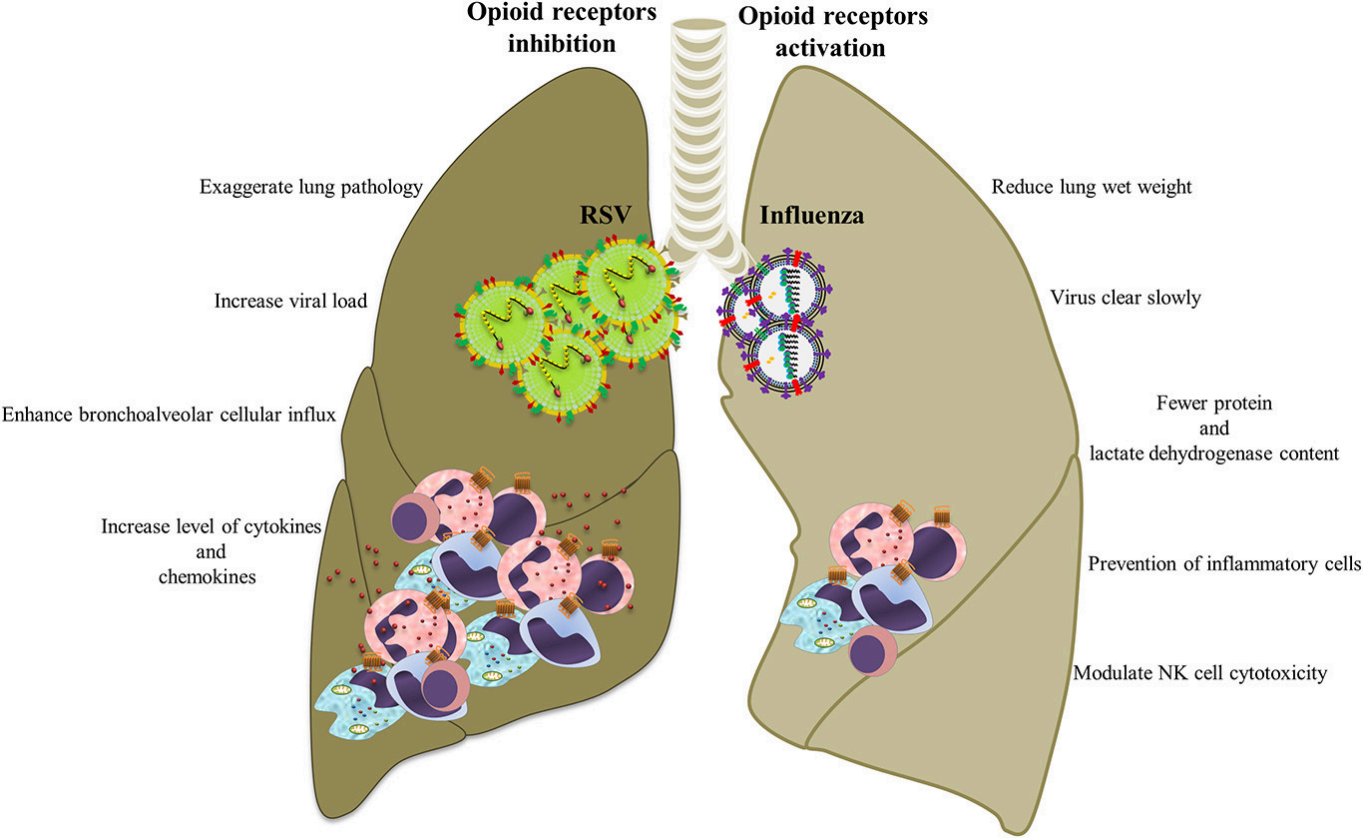
**Opioid systems effects on influenza/RSV immunopathogenesis**. Opioids may attenuate immunopathogenesis of influenza and RSV via immunomodulatory and anti-inflammatory actions.

Because opioids alter immune function, it is likely that this system also effects the immunopathogenesis of influenza in the pulmonary tract (Figure 5). Coussons-Read et al. examined the effect of morphine treatment on pulmonary inflammatory response to intranasal influenza infection in Lewis rats (Coussons-Read et al., 1999). They show that morphine impairs the inflammatory response to influenza infection in the lung by prevention of inflammatory cells in the lung, lowering the percentage of polymorphonuclear cells in the bronchoalveolar lavage fluid (BALF), decreasing the protein and lactate dehydrogenase content in BALF, and reducing the wet weight of the lung (Coussons-Read et al., 1999). They also revealed that the influenza virus cleared slowly from the lungs of morphine-treated rats compared to non-treated and placebo controls, suggesting that there may be a relationship between impaired inflammatory response and viral clearance (Coussons-Read et al., 1998).

Tseng et al. revealed that opioid systems activate MORs which modulate NK cell cytotoxicity during influenza infection (Tseng et al., 2005). Importantly, opioids could increase the risk of pneumonia after influenza as a consequence of immune suppression function (Dublin et al., 2011). Chen et al. showed that exposure to methadone significantly increased H1N1 viral replication in lungs, which might increase the augmented risk of serious influenza A virus infection in people who receive opioid-dependence therapy (Chen et al., 2015).

These results suggest that the opioid systems impair or modulate immune responses induced by the influenza virus that might be beneficial for controlling viral immunopathogenesis, but may lead to delayed viral clearance. Although the data clearly show that opioid system alters the pulmonary inflammatory response to influenza infection, the mechanism of these effects remains unclear. An important step in understanding the role opioids play in pulmonary immune status is unraveling the underlying mechanism of the effects of opioid system on pulmonary inflammation.

### Respiratory syncytial virus

Respiratory syncytial virus (RSV) causes severe respiratory disease and even death in neonates and the elderly (Lozano et al., 2012; Salimi et al., 2016). RSV infections such as influenza causes infiltration of immune cells by infected epithelial cells, which causes immunopathology (Stoppelenburg et al., 2013). Opioids are frequently used during mechanical ventilation of severe RSV infection and some studies have examined the effects of opioids and their receptors on RSV infection. A human study and an experimental model by the authors recently demonstrated opioid system control of RSV replication in the lung and of virus-induced pathology (Figure 5). In the human study, A118G single nucleotide polymorphism rs1799971 in the OPRM1 gene was found to be associated with RSV disease severity. Cases with G alleles are less prone to develop severe RSV infection than those with A alleles (Salimi et al., 2013). A single nucleotide polymorphism at position 118 in the coding region of the OPRM1 alters immune response and prevents secretion of pro-inflammatory cytokines such as IL-6, IL-8, and TNF-α (Matsunaga et al., 2009). In mice, administration of the opioid receptor antagonist nalmefene increased RSV viral load and is associated with increased levels of cytokines and chemokines in the BALF, enhanced bronchoalveolar cellular influx, and exaggerated lung pathology. It was also demonstrated that activation of opioid receptors using μ (DAMGO), κ (U50488), and δ (DPDPE) agonists reduced neutrophil influx (Salimi et al., 2013).

Taken together, these results suggested that opioid systems have a beneficial role in the outcome of respiratory viral disease and could serve as effective therapeutic targets. Opioid receptor activation could offer powerful novel pharmacologic targets to amend RSV-induced immunopathogenesis, although the clinical effects of pharmaceutical agonists should be evaluated.

### Herpes simplex virus

Herpes simplex viruses (HSVs) are a worldwide infectious agents causing variety of diseases such as oral and genital lesions, encephalitis, neonatal infections, and tumors (Whitley and Roizman, 2001). Opioids administration is known to affect the course of HSV infection because of their immunosuppressive function. For example, morphine, a broadly studied and widely used opioid, is known to alter HSV infection, immune response, as well as latency (Jamali et al., 2007a, 2012; Mojadadi et al., 2009; Bauchat, 2010).

It has been clearly demonstrated that morphine alters innate immune responses, which affect the ability of an infected host to mount a specific immune response and clear HSV-1 (Sheridan and Moynihan, 2005). Acute morphine administration diminishes the ability of white blood cells to induce a protective innate immune defense against HSV-1 infection through inhibition of IFN-γ production and NK cell activity (Jamali et al., 2007a). Mojadadi et al. found that acute morphine administration significantly reduced cytolytic T lymphocyte response, lymphocyte proliferation, and IFN-γ production, which consequently reactivated HSV-1 latency in BALB/c mice (Mojadadi et al., 2009). Cellular immune response plays an important role in the inhibition of HSV reactivation and withdrawal from morphine has been shown to reduce protective immunity against acute HSV-1 (Jamali et al., 2012). Endogenous opioids suppress protective immunity against primary HSV-1 infection (Jamali et al., 2007b). Paradoxically, morphine treatment reduced HSV-1-mediated pathology in infected mice by alleviating some of the unwarranted immunopathological manifestations associated with these infections, but not by reducing the viral replication rate (Alonzo and Carr, 1999; Weeks et al., 2001).

Given the immunosuppressive effects of opioids, it has been demonstrated that morphine reactivates latent HSV (Davies et al., 2005). Gieraerts et al. reported high prevalence of HSV infection in patients receiving epidural morphine following cesarean section (Gieraerts et al., 1987). A significant association between the use of epidural morphine which is commonly used for postoperative analgesia after cesarean section and reactivation of oral herpes in the obstetric population has also been reported (Crone et al., 1990).

Acute morphine administration potentiates the development of encephalitis in HSV-1 infected mice (Lioy et al., 2006). In this regard morphine exposure decreases integrity of the blood-brain barrier (BBB) and might explain its potential role for involvement of BBB in the development of encephalitis in morphine-treated mice (Lioy et al., 2006). It has been shown that a hippocampal dynorphin system causes seizures in HSV-1 infected rats. Dynorphin is the preferred endogenous opioid for KOR activation with broad homeostatic functions (Solbrig et al., 2006). Furthermore, activation of peripheral MORs by morphine and loperamide suppresses mechanical allodynia in mice with herpetic pain (Sasaki et al., 2007, 2008).

Altogether, these findings indicate that opioid systems suppress protective immunity against HSV infection and induce HSV reactivation. More extensive studies are required to explore the role of opioid system in HSV and other herpes viruses such as EBV and CMV infection for more efficient therapeutic approaches.

## Conclusion

In recent years, data the opioid system has received attention because it demonstrates the influence on immune cells and immune function. In addition, there is a large body of evidence from *in vivo* and *in vitro* models indicating that opioid systems effect viral replication and virus-mediated pathology. The diverse effects of the opioid system on viral infection imply involvement of different mechanisms. The effect of opioids on immune cells, epigenetics, viral, and cellular factors are implicated in the pathogenesis. For the majority of viruses, opioid system enhances viral pathogenesis by modulation of immune responses (Sheridan and Moynihan, 2005; Wang X. et al., 2005; Zhang et al., 2006). Accordingly, blockage of opioid receptors can potentially be applied for viral control. On the other hand, activation of opioid receptors is beneficial for controlling viral pathogenesis in those viral infections where host immune responses are pathogenic (Coussons-Read et al., 1999; Salimi et al., 2013).

Exploring the interplay between viruses and opioid system and corresponding molecular mechanisms is an important tool for understanding the effect of opioids, mechanisms of action, and development of therapeutic strategies. Clearly more work needs to be done, especially in clinical settings, to promote the importance of understanding the exact interaction between opioid system and viral infections. Because endogenous opioid systems have favorable functions, manipulation of these systems should be performed with caution. To investigate this interaction, further research is required in the following areas:

- While opioids and their receptors are involved in the pathogenesis of several viral infections, the exact mechanisms remain unknown. For example, the signaling pathways between opioids and viruses require further investigation.
- Although the *in vitro* and animal model effects of opioids are well documented, few have explored the effect of opioids on viral infection in clinical settings and future studies with a larger sample sizes are necessary to determine the opioid system effects.
- Initial data indicates a beneficial role of opioids in the outcome of respiratory viral diseases. Novel essential therapeutic approaches based on opioid system activation and/or inhibition are required for full characterization.

## Author contributions

Conceived and designed: AT, MT-Y, VS. Analyzed the data: AT, MT-Y, TM, VS. Wrote the paper: AT, MT-Y, TM, MT-R, LB, FS, VS. Helpful discussions and critical review of the manuscript: TM, LB, FS.

## Funding

This work was partially supported by the School of Public Health, Tehran University of Medical Sciences (28054) and University Medical Centre Utrecht (R1763). The funders had no role in study design, data collection and analysis, decision to publish, or preparation of the manuscript.

### Conflict of interest statement

The authors declare that the research was conducted in the absence of any commercial or financial relationships that could be construed as a potential conflict of interest.

## Abbreviations

MORs: μ-opioid receptors
DORs: δ-opioid receptors
KORs: κ-opioid receptors
GPCRs: G-protein-coupled receptors
MAPK: mitogen-activated protein kinases
IP3, inositol-1, 4: 5-triphosphate
CCR: C-C chemokine receptor
CXCR: C-X-C chemokine receptor
CXCL: C-X-C motif ligand
CCL: C-C motif ligand
HIV: human immunodeficiency virus
Th: T-helper
CNS: central nervous system
miRNAs: microRNA
3′-UTR: 3′-untranslatet region
ROS: reactive oxygen species
NF-κB: nuclear factor-kappa B
gp: glycoprotein
Tat: transactivator of transcription
TNF-α: tumor necrosis factor-α
IL: interleukin
HCV: hepatitis C virus
IFN: interferon
IRF: interferon regulatory factor
SOCS-3: suppressor of cytokine signaling 3
PIAS-3: protein inhibitor of activated STAT3
NO: nitric oxide
BALF: bronchoalveolar lavage fluid
RSV: respiratory syncytial virus
HSV: herpes simplex virus
BBB: blood-brain barrier.
